# Reports on the Hospitals of the United Kingdom

**Published:** 1911-12-02

**Authors:** Henry Burdett


### REPORTS ON THE Hospitals of the United Kingdom.

By SIR HENRY BURDETT, K.C.B., K.C.V.O.

### Johnson Hospital, Spalding.—II.

No. LVII of these reports (The Hospital, November 18, 1911, p. 183) dealt with the above hospital. It contained two verbal errors which we may first correct. In the first column, line five, for the word "modern" substitute "endowed," and in line fourteen for the word "institute" substitute "institution." With this exception we must adhere to this report as faithfully recording the actual condition of the institution as we found it on the day of our inspection, September 4, 1911. The trustees, with whom of course we have no acquaintance, nor any personal knowledge, who, we are quite prepared to believe, are well-meaning and excellent gentlemen individually, have evidently failed to realise their responsibility as trustees for a public hospital in the present day, as the reply to our report, which they have handed to the Press, forcibly illustrates. They have not sent us any reply, and we are indebted to our contemporary, the *Nottingham Guardian*, of November 21, for the statement handed to their reporter on behalf of the trustees on the previous day.

### THE TRUSTEES' REPLY.

We take the following from the *Nottingham Guardian*, November 21, 1911:—

The Johnson Hospital trustees held a special meeting yesterday afternoon (November 20, 1911) to consider Sir Henry Burdett's criticism. The Rev. Canon Bullock presided over the meeting, which was fully attended, and was held in private.

At 3.50 the following statement was handed to our representative:

"The trustees met by special meeting to consider the unwarrantable attack made upon them in The Hospital by Sir Henry Burdett. It should be noticed that Sir Henry is both editor and proprietor of The Hospital, but it is difficult to understand why he should go out of his way to make so violent an attack upon the trustees of the Johnson Hospital in regard to the care taken of it.

"After discussing the whole matter thoroughly, the following resolutions were passed:—

"1. That no application for permission to view the hospital, nor any notice of any intended visit, was ever made to any one of the trustees.

"2. That had Sir Henry Burdett discussed the situation with the trustees, who, he knew, would meet within half an hour of his quitting the premises, explanations would have been forthcoming, which would have prevented undoubtedly the publication of his extravagant inaccuracies.

"3. With regard to the main strictures of the report, the trustees indignantly repudiate the misstatements concerning the structural defects of the hospital. The outside of the building is in perfectly good condition, and was quite recently painted throughout. The inside has been kept in the best repair that the income at the disposal of the trustees would permit of, and was partly redecorated as lately as the spring of this year.

"4. That the hospital has met with the entire satisfaction of the patients, so far as the trustees know."

Taking the paragraphs of this document in order, it will be observed that the trustees in the first paragraph describe our report as "the unwarrantable attack," which we "have gone out of our way to make." The trustees are wrong on both points, for the report contains no attack, unwarrantable or otherwise, and was made in connection with an inspection of the voluntary hospitals throughout the country which was begun in the autumn of 1909, and is still proceeding. When it was begun these inspections were welcomed by the Press because they were faithfully and clearly to report the actual condition of each hospital as we found it to be.

We can fearlessly maintain that we have observed this spirit throughout our inspections, which have been welcomed by the best administered hospitals all over the country, and have proved most fruitful for good to the hospitals and in other ways. Our object has been, and is, to record without fear or favour the actual state of affairs as we found it at each hospital at the time of our visit, and to indicate, where necessary, the directions in which it may fail, if at all, to attain to the recognised standard of a modern hospital. We went to Spalding on purpose to see the Johnson Hospital, because we visited it when it was first opened, remembered the lavish way in which the buildings had been designed and executed, and were anxious to ascertain whether or not this modern endowed hospital was being maintained in a state of efficiency. Our report is an accurate record of the actual condition of the hospital as we found it. The condition of this hospital and its state of internal neglect, to which our remarks were practically confined, were in fact unjustifiable.

The trustees complain:—(1) That no application for permission to view the hospital nor any notice of any intended visit was ever made to any one of the trustees. Could anything better illustrate the unfortunate position into which the affairs of this public institution have fallen? for it is the glory of the voluntary hospitals of this country that, they are at all times open to the inspection of any one interested in them. We have visited all the principal hospitals throughout Europe, the United States, and several portions of the British Empire, and never once has a similar complaint been made by a reputable hospital. The truth is that to be effective an independent inspection must be made without notice, so that the actual condition of the hospital and the atmosphere in which the work is being done may be accurately ascertained and impartially recorded.

(2) Complaint is made that Sir Henry Burdett "did not discuss the situation with the trustees who he knew would meet within half an hour." Sir Henry Burdett had no such knowledge, and, had he possessed it, he would have welcomed the opportunity of taking the trustees personally through their own hospital, and pointing out to them the actual condition of affairs which even now their reply indicates they do not realise.

(3) The trustees indignantly repudiate the statement concerning the structural defects of the hospital. Instances of the structural defects which actually exist are given in our report, and, to be effective, any reply on the part of the trustees must indicate and prove which of these they hold to be misstatements. For our part, we invite the trustees, before they proceed to make any alterations or changes, to throw open the whole institution to the inspection of the Press and the public, that they may see the actual internal and structural neglect in which we found the hospital on the day of our visit. We accept the statement that the outside of the buildings has been quite recently painted, but a reference to our report will show that our criticisms were confined to the inside conditions, though we urged, and still hope, that the whole buildings will be overhauled from top to bottom, inside and out.

The trustees state the inside has been kept in the best repair that the income at the disposal of the trustees would permit of. A reference to the revenue account printed in the thirtieth report for the year ending December 31, 1910, shows that the financial condition of the hospital is as follows:—The total expenditure under all heads amounted to £922 19s. 10d.; the income derived from invested funds produced £850 12s. 8d.; the income derived from patients' payments amounted to £63 10s. 6d., and £8 was derived from the rental, presumably, of a portion of the buildings, making a total income of £922 3s. 2d. Further, the trustees invested £300 in the purchase of Consols, and had in hand in cash at the beginning of 1911, £155 14s. 7d. The plea of want of funds is not, therefore, maintainable, seeing that needed repairs should not exceed, if they even amounted to, a sum of £250, and that the money which was actually in hand and available was nearly double that sum, and so will leave an ample margin in hand. Further, the figures quoted show that the actual income derived from invested property and patients' payments, exclusive of the subscriptions, donations, alms boxes, &c., which we have not included, are ample to defray the cost of running the hospital as it is at present conducted, and to keep it in every way efficient and up to date as a modern hospital.

### What are the Reasons for the Present Neglect?

We hope we have demonstrated that there is no substance in the reply of the trustees, and that the interests of the poor of Spalding and the district who use the hospital demand that they shall at once proceed to put this hospital in the most perfect order from top to bottom. We were careful to say in our report that it was not necessary for us to find the reason for the present neglect. How much may the facts stated in the following question put to us by the *Spalding Guardian* have to do with the matter? "What Sir Henry may not know is that the trustees are a close corporation of well-meaning but conservative gentlemen who refuse to permit the smallest element of popular representation. This is the usual consequence. Another consequence is the almost complete alienation of public interest and sympathy." We of course had no knowledge of the facts here stated, but our report shows that we felt from the actual atmosphere of the place that there must be almost complete alienation of public interest in and sympathy with the hospital in its work. Again, is the explanation to be found in the statement contained in the *Spalding Free Press*? which, in a leading article, states: "We by no means approve of the constitution of the governing body, formed as it is on such exclusive lines. . . . There can be little question that the selection of the trustees from one particular class is against the best interests of the institution. It decidedly lessens the public interest in the welfare of the hospital, and there can be no doubt that it materially reduces the amount of public subscriptions, which certainly stands at a rather insignificant sum. The governing body consists entirely of members of the Church of England. In the thirty years we can only recall one Nonconformist trustee. We are not suggesting that the question of creed has made any difference in the reception or the treatment of patients—we do not believe for a moment that it has . . . but in the management of public institutions, an exclusive policy is an undesirable one, and is especially indefensible where an appeal is made to the public for support."

### A Hint to Spalding.

The writer of these reports is the son of a clergyman of the Church of England, and his uncle was for many years rector of a Lincolnshire parish not far from Boston; but an experience of forty-five years in the administration of hospitals and charities generally, and of great undertakings, compels him to uphold the justice of the points made by the *Spalding Free Press*. We have no quarrel with the trustees, who are strangers to us, and whose names we do not even know. But in this matter we have acted as the representative of the public outside the hospital. Therefore, although we hope the trustees may even at the eleventh hour recognise it to be their duty, immediately, to put the hospital throughout in as perfect a condition for hospital purposes as possible, we must point out, too, that the responsibility henceforth for the condition of the Johnson Hospital, Spalding, must be shared by the people of Spalding and the neighbourhood, for whose advantage it was founded by two benevolent ladies. We hope that some one in authority in Spalding will summon a representative meeting of inhabitants to appoint a deputation to confer with the trustees, with the object of securing the needed reforms in the constitution as well as in the actual condition of this hospital. In any case the machinery of the Charity Commissioners is quite adequate to deal with this matter and to bring about these reforms with assured certainty.

